# Intrapericardial delivery of visible microcapsules containing stem cells using xfm (x-ray fused with magnetic resonance imaging)

**DOI:** 10.1186/1532-429X-13-S1-P26

**Published:** 2011-02-02

**Authors:** Nicole M Azene, Tina Ehtiati, Yingli Fu, Aaron Flammang, Jens Guehring, Wesley D Gilson, Dorota Dorota Kedziorek, Judy Cook, Peter V Johnston, Dara L Kraitchman

**Affiliations:** 1 Johns Hopkins University School of Medicine, Baltimore, MD, USA; 2 Siemens Corporate Research, Baltimore, MD, USA

## Introduction

Previously, we have demonstrated a technique to enhance survival of transplanted cells using XFM-visible microcapsules. However, theoretical concerns exist about the induction of arrhythmias if microcapsules are delivered to the heart by transendocardial injection. Delivery of these microcapsules to the pericardial space may provide an alternative approach with less potential for arrhythmia. Pericardial approaches to the epicardium have been used for the delivery of cardioactive drugs, cardiac ablation techniques and the implantation of cardiac pacemakers. However, there are no reports involving the utilization of the pericardial space for stem cell delivery.

## Purpose

This study tests the feasibility and accuracy of an intrapericardial delivery approach of barium-alginate microcapsules (Ba-X-Caps), a vehicle for allogenic mesenchymal stem cells (MSCs) implantation.

## Methods

Five Yorkshire pigs underwent a whole heart 3D TrueFISP navigator-gated cardiac MR (1.5T Espree, Siemens, 209 msTR; 1.6 msTE; 320x240 mm FOV; 256x172 image matrix; iPAT=2; 2 mm slice thickness, 64 slices) followed by cardiac-gated c-arm CT of the heart (DynaCT, Axiom Artis dFA, Siemens, 190° rotation; 0.5° angle; 20s acquisition; 48 cm FOV). After 3D-3D registration of the MRI and c-arm CT, a segmented MRI of the epicardial and endocardial sufaces was volume-rendered and overlaid on the c-arm CT and live X-ray fluoroscopic imaging (Figure).

Pericardial access was achieved with a 17 g Touhy epidural needle via a percutaneous subxiphoid approach under XFM guidance (iPilot, Siemens) taking care to avoid the coronary vasculature. Needle placement was confirmed by a small injection of iodinated contrast. The epidural needle was exchanged over a guide wire for a 4F introducer sheath followed by Ba-X-Caps (7-10cc) administration into the pericardial space. For chronic studies (7 days post-injection), an echocardiogram was performed to assess cardiovascular function and pericardial integrity. C-arm CT was then performed to assess Ba-X-Cap persistence. After humane sacrifice, the heart and Ba-X-Caps were harvested for histological evaluation.

## Results

**Figure 1:**
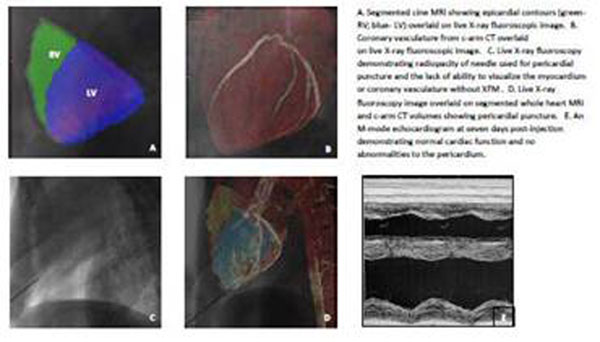


We were able to successfully transplant Ba-X-Caps in 80% of the pigs using XFM-guidance (Figure 1). Transplanted Ba-X-Caps were visualized in the epicardium of pigs surviving 7 days post-injection. Pericardial adhesions did not develop in any of these animals (Figure 1). Cardiac function was also preserved (LVEF = 64.74% ± 1.24).

## Conclusions

XFM-guided intrapericardial delivery of Ba-X-Caps is feasible in swine and provides a safe, minimally-invasive method for X-ray-visible stem cell administration. Short-term follow-up of intrapericardial administration of Ba-X-Caps shows a good biocompatibility profile.

